# Salivary characteristics and dental caries experience in remote Indigenous children in Australia: a cross-sectional study

**DOI:** 10.1186/s12903-018-0692-2

**Published:** 2019-01-17

**Authors:** R. Lalloo, S. K. Tadakamadla, J. Kroon, O. Tut, S. Kularatna, R. Boase, K. Kapellas, D. Gilchrist, E. Cobbledick, J. Rogers, N. W. Johnson

**Affiliations:** 1 School of Dentistry, The University of Queensland, Brisbane, Australia; 2 Menzies Health Institute Queensland, Griffith University, Gold Coast Campus, Gold Coast, Queensland 4222 Australia; 3 School of Dentistry and Oral Health, Griffith University, Gold Coast, Australia; 4 Australian Centre for Health Service Innovation, School of Public Health and Social Work, Institute of Health and Biomedical Innovation, Queensland University of Technology, 60 Musk Avenue, Kelvin Grove, QLD 4059 Australia; 5 School of Dentistry, James Cook University, Cairns, Australia; 6 Australian Research Centre for Population Oral Health, School of Dentistry, The University of Adelaide, Adelaide, Australia; 7 King’s College London Dental Institute, London, UK

**Keywords:** Dental caries, Saliva, Bacteria, Children, Fluoridation, Remote, Indigenous, Australia

## Abstract

**Background:**

While associations between salivary characteristics and dental caries have been well studied, we are not aware of this being assessed in a remote Indigenous child population, where lifestyles may be different from urban children. Our aim was to assess associations between caries experience and putative biomarkers in saliva, accounting for oral hygiene and dietary habits.

**Methods:**

Children attending schools in an Indigenous community in remote north Queensland, Australia were invited to an oral examination by qualified and calibrated examiners. Salivary flow rate, pH, buffering capacity and loads of mutans streptococci (MS), lactobacilli (LB) and yeasts were determined. Also, data on tooth brushing frequency and soft drinks consumption were obtained via a questionnaire. Caries experience was recorded by the International Caries Detection and Assessment System (ICDAS-II), and quantified as decayed, missing and filled surfaces. Relationships between the salivary variables and the cumulative caries experience (dmfs+DMFS) in the deciduous and permanent dentitions were examined by multivariate analyses to control the effect of confounders.

**Results:**

The mean cumulative decayed (DS + ds), missing (MS + ms) and filled (FS + fs) surfaces were 3.64 (SD: 4.97), 1.08 (4.38) and 0.79 (1.84) respectively. Higher salivary MS and LB counts, low tooth brushing frequency and daily soft drink consumption were significantly related to greater caries experience. Caries experience was about twice in those with ≥10^5 CFU/ml saliva counts of MS (mean = 6.33, SD: 8.40 vs 3.11, 5.77) and LB (7.03, 7.49 vs 4.41, 8.00). In the fully-adjusted multivariate model, caries experience in those with higher counts of MS and LB were 51 and 52% more than those with lower counts.

**Conclusions:**

As with studies in other populations, childhood salivary counts of MS and LB were significantly associated with greater caries experience in this remote Indigenous community. To address the serious burden of oral disease, we are researching ways to promote a healthy oral environment by encouraging good dietary habits, and emphasising the importance of daily tooth brushing with a fluoridated toothpaste. Our ongoing longitudinal studies will indicate the success of measures employed to reduce the counts of bacteria closely associated with cariogenesis and their impact on caries increment.

**Trial registration:**

Australian New Zealand Clinical Trials Registry (ANZCTR), No: ACTRN12615000693527; date of registration: 3rd July 2015.

## Background

Worldwide amongst the fifty most common chronic diseases, four occur in the mouth: (1) dental caries of permanent teeth (ranked 1st), (2) chronic periodontitis (ranked 6th), (3) dental caries of deciduous teeth (ranked 10th), and (4) edentulousness (total tooth loss, ranked 36th) [1, 2]. These are highly prevalent and severe in Indigenous communities in Australia, constituting a particularly heavy burden in remote communities [3, 4]. Compared to national averages, Indigenous children in Australia living in rural and/or remote parts of the nation have much higher mean numbers of decayed, missing and filled teeth in the deciduous dentition (dmft) (~ 4 in 6-year old children) compared to non-Indigenous children in both metropolitan (dmft ~ 1.5) and rural communities (dmft ~ 1.8) as well as Indigenous metropolitan children (dmft ~ 2.6) [5]. This is also true of the permanent dentition of older children. This incurs significant social costs, and poor quality of life due to pain and discomfort. Poor sleep, time off school, and disturbed behaviour contribute to poor learning [6–9].

Saliva is protective of the oral hard and soft tissues as it facilitates clearance of food debris and sugar, contributes to aggregation and elimination of microorganisms, has a buffering capacity to neutralise acids, promotes remineralisation of tooth enamel and has other antimicrobial properties [10, 11]. Thus, salivary composition such as microorganisms, and functional properties such as flow rate have been found to be associated with dental caries to varying degrees [10]. Such salivary properties can be used as biomarkers for risk of future disease and could potentially inform interventions to address this risk [10–13].

While the associations between these salivary characteristics and dental caries have been well-studied [14–17], we are not aware of this being assessed in a remote Indigenous child population in Australia. To address the significantly greater burden of dental caries in this population it is important to investigate risk indicators for caries to inform prevention and health promotion policies and interventions. Whilst many studies show that the strongest predictor of future caries is past caries experience [18], a recent comprehensive review [19] has shown that the utility of biological caries risk predictors differ between countries. Remote Indigenous communities in Australia may have a different mix of risk indicators than elsewhere in Australia, and these may have aspects in common with other Indigenous communities around the world. The objective of this study is, therefore, to assess the associations between dental caries experience and salivary flow rates, salivary pH and buffering capacity and the salivary load of certain bacteria, accounting for oral hygiene and dietary habits. Bacteria traditionally measured in relation to dental caries are mutans streptococci [MS], lactobacilli [LB] and yeasts. Such measures are frequently advocated by the dental profession and there are several commercial kits for their measurement [20].

## Methods

### Study design, study setting and participants

**Fig. 1:**
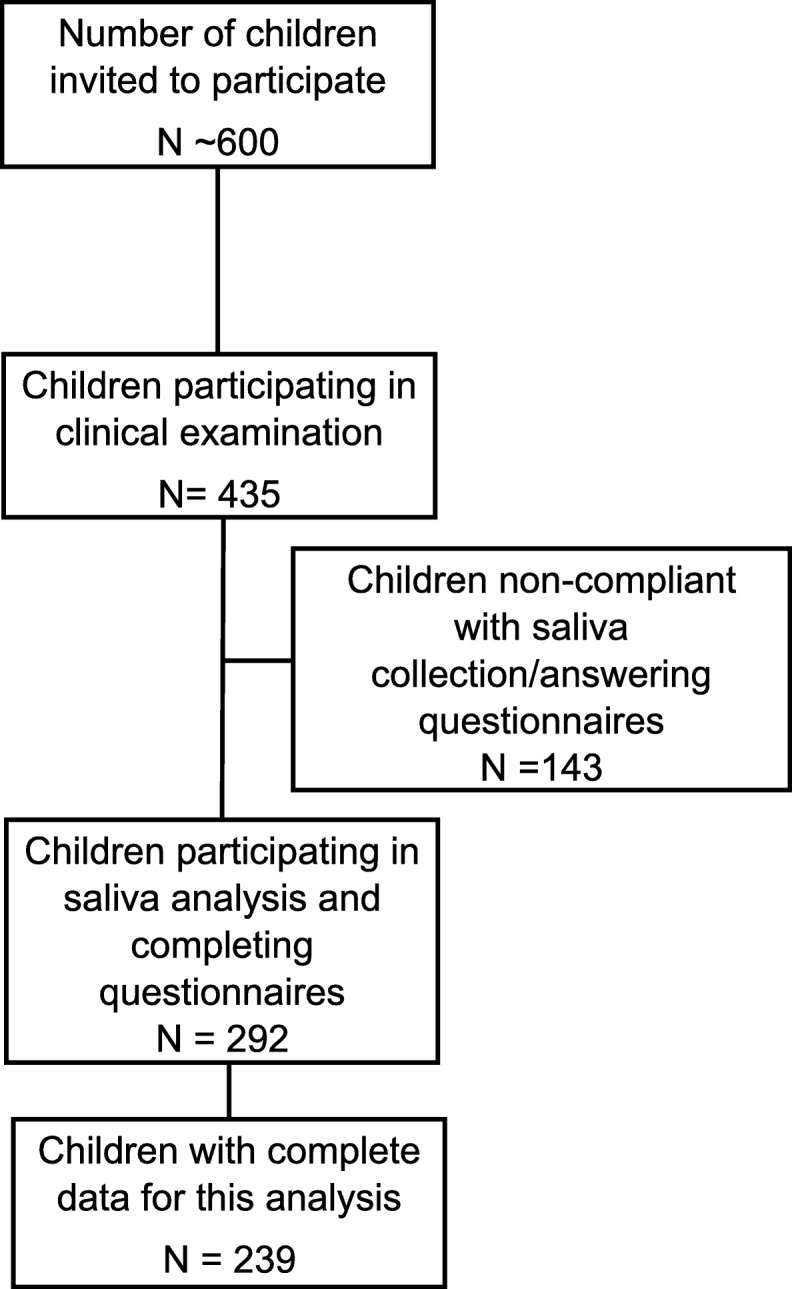
Number of children approached and those that consented, and complied, with particular aspects of the study

The overarching aim of the longitudinal study is to assess the effectiveness, cost-effectiveness and cost-benefit of a single annual professional intervention for the prevention of childhood dental caries in a remote rural Indigenous community. The analysis presented here comes from the baseline cross-sectional data of this intervention study. The community is located in remote north Queensland, Australia and is 1000 km from the nearest regional city, which has a population of ~ 161,000 [21]. The study community has a population of approximately 2000 persons. All children (nominally ~ 600) enrolled at the two primary and one secondary schools in the community were invited to participate in our study prior to the visit of the research team. A community member was employed for a number of months in 2015 to assist with the recruitment of children for the study. These children are between 4 and 17 years of age, and almost all are Aboriginal or Torres Strait Islander people (Indigenous). Of the about 600 children, 435 participated in the clinical examination phase of the study and 292 provided a saliva sample (Fig. 1). Data collection occurred over a number of weeks in the second half of 2015.

### Outcome variable - dental caries

The dental caries status of the children was assessed by three trained and calibrated examiners. We used ICDAS-II, the International Caries Detection and Assessment system [20]. Children were examined in a classroom, using a mobile, reclinable dental chair with fixed- and head-LED lights. Disposable mouth mirrors and blunt probes were used, and gauze used to control moisture. For this analysis caries experience was defined as the sum of tooth surfaces with an ICDAS-II caries code of 4 to 6 (decayed), plus missing and filled surfaces in both the deciduous and permanent dentitions (whole mouth): dmfs+DMFS. A child was considered to experience caries if he/she had at least one decayed, missing or filled surface in the mouth: dmfs+DMFS> 0. All examiners completed the ICDAS-II online training module prior to the community visit. To assess inter-examiner reliability, agreement was tested using Kappa statistics. Approximately 5% of children were re-examined by the three examiners; and discrepancies in scores were discussed with the child present. The overall Kappa was 0.837, indicating a high level of agreement between the examiners.

### Exposure variables – Salivary characteristics

Prior to the oral screening examination, a sample of stimulated saliva was collected over 5 min using commercially available kits for recording flow rate, pH and buffering capacity [GC Corporation, <http://www.gcaustralasia.com/Products/97/Prevention/Saliva-Check-BUFFER]. To assess saliva flow rate, the child was asked to chew on a piece of wax for 5 min, expectorating into a collection cup every 30 s or sooner if they felt more comfortable. The volume of saliva collected after 5 min was recorded in millilitres. To measure pH, the litmus strip from the GC SalivaCheck test kit was dipped into the cup and the pH recorded after 30 s. For buffering capacity, three drops of saliva were placed onto the three GC test pads using the pipette provided, left for 5 min, and the colour matched against the manufacturer’s chart, and the three recordings added. A high count of bacteria in dental biofilm correlates with salivary bacterial counts, enabling saliva as a source for quantifying cariogenic bacteria [22, 23]. These commercial kits use selective media for MS and LB: these media also support the growth of oral fungi, detectable by their large colony size.

(http://www.ivoclarvivadent.com/en/p/all/products/prevention-care/caries-risk/crt-bacteria) [24]. CRT agar plates were coated with saliva, following manufacturer’s instructions, and incubated for 48 h at 37 °C. The number of colony forming units was estimated by referring to the CRT charts. Bacterial counts were recorded as follows for MS and LB: < 10^5 CFU/ml saliva or ≥ 10^5 CFU/ml saliva. Yeast counts were categorised into none/light and moderate/heavy.

### Other explanatory variables

Age, gender, tooth brushing frequency and daily soft drink consumption information were collected via a questionnaire. Specifically, children were asked the frequency of daily tooth brushing; categorised into ‘Once or less’ and ‘Twice or more’ for this analysis. They were asked if they consumed a soft drink on a typical day (Yes or No). The effect of these variables was controlled in the fully-adjusted multivariate analysis as they could serve as confounders.

### Statistical analysis

SPSS 24.0 (IBM, New York) was used for conducting the statistical analyses. Descriptive statistics were performed to evaluate the mean decayed, missing and filled surfaces for deciduous and permanent dentitions together (caries experience = dmfs+DMFS). Caries experience (dmfs+DMFS) was the outcome variable in this study. As caries experience was not normally distributed, Mann Whitney U test was used to compare the caries experience in relation to gender, salivary physiology and microbiology, and a selection of behavioural characteristics. In this part of the analysis a number of continuous explanatory variables were dichotomised: salivary pH (<=6.6 and > 6.6); flow rate (<=5 ml/5 min and > 5 ml/5 min) and buffering capacity (<=9 and > 9) (Table 1). The mean (SD), median (interquartile range; IQR) and *p*-value for the Mann Whitney U test are reported. With the count data (dmfs+DMFS) widely dispersed (variance was greater than the mean), a Generalised Linear Model with negative binomial regression and log link was used to explore the association with demographic (age and gender), salivary physiology (pH, salivary flow rate and total buffering capacity as continuous variables), salivary microbiology (MS, LB and Yeast counts) and behavioural characteristics (tooth brushing frequency and soft drinks consumption on a typical day). In order to observe the effect of each explanatory variable separately, univariate analyses was conducted by entering one variable at a time. This was followed by a fully-adjusted multivariate model where all the variables were entered at once. Exponential estimates of the negative binomial regression analyses are presented as Prevalence Rate Ratios (PRR) with 95% confidence intervals (95% CI). For all tests conducted, a p-value of < 0.05 was considered statistically significant.

**Table 1:** Frequency distribution of gender, salivary and behavioural characteristics

	N (%)
Gender^a^	
Male	128 (44.6%)
Female	159 (55.4%)
Salivary pH^a^	
≤ 6.6	45 (15.6%)
> 6.6	244 (84.4%)
Salivary flow rate (ml/5 min)^a^	
≤ 5	137 (47.7%)
> 5	150 (52.3%)
Total buffering capacity^a^	
≤ 9	151 (53%)
> 9	134 (47%)
Salivary MS counts	
≥ 10^5 CFU/ml saliva	212 (72.6%)
< 10^5 CFU/ml saliva	80 (27.4%)
Salivary LB counts	
≥ 10^5 CFU/ml saliva	116 (39.7%)
< 10^5 CFU/ml saliva	176 (60.3%)
Salivary Yeast counts^a^	
Moderate or heavy	44 (15.3%)
None or light	243 (84.7%)
Brushing frequency^a^	
≤ once/day	63 (24.3%)
>2 day	196 (75.7%)
On a typical day, do you consume soft drink^a^	
Yes	204 (77.3%)
No	60 (22.7%)

^a^Some participants had missing data, so the categories do not add up to total sample size

## Results

Of the 435 children who underwent the clinical examination and answered the questionnaires, 292 had a MS and LB colony count recording (Fig. 1). A number of children were unable to or were not compliant in providing a salivary sample for analysis. There were some subjects who had missing data for a few variables, and the sample size included in the multivariate analysis was 239. However, in the univariate and bivariate analysis, all the children with data for a specific variable were included.

The mean age of the children was 8.79 (SD: 3.45), there were more females (55.4%) than males. A majority of children (84.4%) had salivary pH of > 6.6 (Table 1). Many children (72.6%) were found to have salivary MS counts of ≥10^5 CFU/ml while salivary LB counts of ≥10^5 CFU/ml saliva were found in 39.7% of the children. Approximately three quarters of the children reported brushing their teeth twice or more a day (75.7%) and consuming soft drinks on a typical day (77.3%).

The mean cumulative decayed (DS + ds), missing (MS + ms) and filled (FS + fs) surfaces were 3.64 (SD: 4.97), 1.08 (4.38) and 0.79 (1.84) respectively. The overall caries experience (dmfs+DMFS) was 5.45 (SD: 7.89). More than three-quarters of children (76.5%) had caries in either of the dentitions (dmfs+DMFS> 0).

Mann-Whitney U test revealed salivary MS and LB counts, tooth brushing frequency and soft drink consumption as significant variables (Table 2). Caries experience was approximately twice in those with higher counts of salivary MS (6.33, SD: 8.40 vs 3.11, SD: 5.77) and LB (7.03, SD: 7.49 vs 4.41, SD: 8.00) than those with lower counts. Children who reported brushing their teeth twice or more a day had significantly lower caries experience (4.80, SD: 7.67) than those brushing less frequently (6.75 SD: 7.82). In addition, caries experience in children reporting consumption of soft drinks on a typical day had a greater caries experience (6.14, SD: 8.74) compared to those reporting less frequent consumption of soft drinks (3.30, SD: 5.34).

**Table 2:** Overall caries experience (dmfs+DMFS) in relation to gender, salivary and behavioural variables

	Mean (SD)	Median (IQR)	Significance^a^
Gender			0.466
Male	5.21 (7.71)	2 (7)	
Female	5.52 (8.10)	3 (6)	
Salivary pH			0.901
≤ 6.6	5.29 (6.79)	2 (9)	
> 6.6	5.52 (8.13)	3 (6)	
Salivary flow rate (ml/5 min)			0.460
≤ 5	5.18 (7.64)	2 (7)	
> 5	5.73 (8.24)	3 (6.75)	
Total buffering capacity			0.391
≤ 9	5.93 (8.85)	3 (7)	
> 9	4.93 (6.85)	2 (5.75)	
Salivary MS counts			**< 0.0001**
≥ 10^5 CFU/ml saliva	6.33 (8.40)	3 (7.75)	
< 10^5 CFU/ml saliva	3.11 (5.77)	1 (3.75)	
Salivary LB counts			**< 0.0001**
≥ 10^5 CFU/ml saliva	7.03 (7.49)	5 (7.75)	
< 10^5 CFU/ml saliva	4.41 (8.00)	1 (5)	
Salivary Yeast counts			0.844
Moderate or heavy	6.36 (10.86)	3 (6.75)	
None or light	5.37 (7.30)	3 (6)	
Brushing frequency			**0.004**
≤ once/day	6.75 (7.82)	4 (8)	
2 > day	4.80 (7.67)	2 (6)	
On a typical day, do you consume soft drink			**0.006**
Yes	6.14 (8.74)	3 (3.75)	
No	3.30 (5.34)	1 (7)	

^a^Mann Whitney U test

In the fully-adjusted model, the expected counts of caries experience in those with salivary MS and LB counts of ≥10^5 CFU/ml saliva were 51% (PRR = 1.51; 95% CI: 1.01–2.25) and 52% (PRR = 1.52; 95% CI: 1.12–2.07) more than those with < 10^5 CFU/ml saliva (Table 3). Caries experience in subjects reporting brushing once or less a day was approximately 47% (PRR = 1.47; 95% CI: 1.04–2.08) higher than those who reported brushing twice or more a day. In the univariate analysis, soft drink consumption was also significantly associated with caries experience. In those reporting not consuming soft drinks on a typical day, the caries experience decreased by a factor of 46% (PRR: 0.54; 95% CI: 0.39–0.74). However, this was only significant in the unadjusted model.

**Table 3:** Negative binomial regression analysis with cumulative (dmfs+DMFS) caries experience as the outcome variable and demographic, salivary and behavioural characteristics as explanatory variables

	Unadjusted (Univariate analysis)			Adjusted (Multivariate analysis)		
Gender	B (SE)	PRR^a^ (95% CI)	P	B (SE)	PRR^a^ (95% CI)	P
Males (*n* = 104)	−0.06 (0.13)	0.95 (0.73–1.22)	0.660	−0.06 (0.15)	0.95 (0.71–1.27)	0.703
Females (*n* = 135)	**Ref**	**Ref**		**Ref**	**Ref**	
Age	0.01 (0.02)	1.01 (0.98–1.05)	0.502	0.005 (0.02)	1.01 (0.96–1.05)	0.820
Mutans streptococci						
≥ 10^5 CFU/ml saliva (*n* = 176)	0.71 (0.15)	2.03 (1.52–2.72)	**< 0.0001**	0.41(0.20)	1.51 (1.01–2.25)	**0.043**
< 10^5 CFU/ml saliva (*n* = 63)				**Ref**	**Ref**	
Lactobacilli						
≥ 10^5 CFU/ml saliva (*n* = 96)	0.47 (0.13)	1.59 (1.24–2.06)	**< 0.0001**	0.42 (0.16)	1.52 (1.12–2.07)	**0.007**
< 10^5 CFU/ml saliva (*n* = 143)				**Ref**	**Ref**	
Yeast						
Moderate or Heavy (*n* = 37)	0.17 (0.18)	1.19 (0.84–1.68)	0.334	0.38 (0.21)	1.46 (0.97–2.18)	0.067
None or light (*n* = 202)				**Ref**	**Ref**	
pH	0.01 (0.14)	1.01 (0.77–1.32)	0.947	−0.08 (0.16)	0.92 (0.68–1.26)	0.615
Saliva flow	0.01 (0.02)	0.99 (0.95–1.04)	0.745	−0.05 (0.29)	0.96 (0.90–1.01)	0.117
Total buffering capacity	−0.06 (0.03)	0.94 (0.88–1.00)	0.054	−0.006 (0.04)	0.99 (0.92–1.07)	0.863
Brushing frequency						
≤ once/day (*n* = 58)	0.34 (0.16)	1.41 (1.04–1.91)	**0.029**	0.39 (0.18)	1.47 (1.04–2.08)	**0.028**
≥ twice/day (*n* = 181)	**Ref**	**Ref**		**Ref**	**Ref**	
Soft drinks						
No (*n* = 54)	−0.62 (0.17)	0.54 (0.39–0.74)	**< 0.0001**	− 0.24 (0.21)	0.79 (0.52–1.20)	0.267
Yes (*n* = 185)	**Ref**	**Ref**		**Ref**	**Ref**	

^a^Prevalence Rate Ratio

## Discussion

High salivary loads of MS and LB were significantly associated with dental caries experience, even after adjusting for other salivary characteristics (pH, flow, buffering capacity), tooth brushing frequency and soft drink consumption. Further, children who brushed their teeth infrequently and consumed soft drinks daily experienced greater caries. Such findings are well known from previous studies [13–17], but have not been previously demonstrated in a remote, Indigenous community.

The relationship between high counts of mutans streptococci and of lactobacilli with high levels of dental caries has long been recognised, and is summarised in the National Institute of Health (NIH) Consensus Conference [25]. Similar results were found in children in rural Kenya, a population somewhat similar to the present one, in that both have little access to dental care – important because dental interventions can significantly alter the oral flora [26]. These associations have also been confirmed in recent studies, for example in a low socioeconomic population in India [27]. Mutans streptococci and several species of lactobacilli are regularly isolated from the surface of carious lesions [27, 28]. These associations are the basis of commercial caries susceptibility tests, such as those used in the present study. These associations do not prove cause and effect, and it is more likely that high counts of these organisms are due to high sugar diets, in which sense they reflect caries risk and are not necessarily the only drivers of the disease process [29]. Indeed modern approaches to microbiomics, involving next generation sequencing methods, imply that complex consortia of bacteria are so associated, and these may be pathogenic [30, 31]. Today, growth of these consortia, composed of commensal species, is regarded as the result of ecological shifts driven by changes in the environment [32, 33], such that a range of ecological approaches to caries prevention are now being explored [34].

Reducing MS colonisation of the mouth at early age, and limiting their contribution to oral biofilms by limiting sugar intake and the practise of good oral hygiene, is important. Inhibiting growth periodically with broad spectrum antiseptics, may help to minimise subsequent dental caries, particularly in a high risk population such as this [35–37]. MS colonisation of the mouth is, however, a complex process and controlling it is difficult [38]. Mother or other carer to child transmission needs to be reduced and there is some evidence that maternal xylitol chewing may reduce transmission of salivary MS [39]. It is however unlikely that a maternal xylitol chewing program in a remote community such as this would be feasible and sustainable.

Approximately three quarters of the children reported brushing their teeth twice or more a day (75.7%). This is more than the national average of 68.5% in children aged 5 to 14 years and 53.5% in Indigenous children [40]. It was observed that those brushing once or less a day had greater caries experience than those claiming to be brushing twice or more a day. It is evident that frequent tooth brushing is associated with lower caries incidence [41]. Australian fluoride guidelines therefore recommend brushing teeth twice every day from 18 months of age [42]. While there is insufficient evidence that school-based interventions to reduce caries are successful in practice [43], provided adequate resources can be found a school tooth brushing program might well be instituted in this community: if attempted it would need to be fully evaluated for its efficacy and cost-effectiveness. Clinical approaches to reducing MS counts, theoretically thereby reducing new caries lesions, include antiseptic mouth rinses and swabs. Tooth resistance can be increased with topical fluoride gels or varnishes [44] and application of fissure sealants on permanent molar teeth: all of the above should be considered in communities with significant caries burden [21]. These approaches require a professional workforce and significant funding, and would be difficult to sustain. Nevertheless, they could prove to be substantially more cost-effective than treating the caries present and in the future. It is the purpose of our present research in this community to conduct a thorough economic analysis of such an approach [21]. Most of these initiatives require a commitment from the individual, family and from the health services: long-term research is needed to assess their feasibility and sustainability. There is likely to be a continuing need for higher level community-based interventions such as community water fluoridation, and attempting to reduce the consumption of sugar and other cariogenic foods, even though health promotion activities such as the latter are notoriously ineffective, and rarely cost-effective [45].

A majority of children (77.3%) were consuming soft drinks on a typical day while the data from Australian National Child Oral Health Survey indicates that half of the Australian population consume a glass or more of sugar sweetened beverages in a day [46]. However, the frequency observed in our study is in accordance with the 73.1% reported for Indigenous children [46]. The Longitudinal Study of Australian children showed that soft drink/cordial consumption increased from 1% at age 1, to 28% by age 2 and 43% by age 10 [47]. This consumption was significantly higher in Indigenous children. We found that caries experience in children consuming soft drinks everyday was approximately twice that of those consuming less frequently. It is obvious now from longitudinal studies and systematic reviews that restricting sugar intake decreases the caries risk [48, 49]. A recent study from Germany modelled the impact of imposing 20% sales tax on sugar-sweetened beverages and concluded that this could decrease caries experience in a low income population [50]; similar findings were observed in a recent Australian study [51].

Based on our findings, there is a need to undertake oral health promotion programs in this population, targeted towards behaviour change. School based oral health promotion programs, including supervised tooth brushing, might assist children in adopting positive oral health-related behaviours. A Cochrane review reported that oral health education in association with supervised tooth brushing was effective in reducing dental caries in children [52]. However, bringing about behaviour change in this population could be a major challenge. A randomised controlled trial that delivered fluoride varnishes along with comprehensive oral health promotion activities has failed to bring change behaviours in young Indigenous children elsewhere in Australia [53]. Although the Queensland Government has been implementing several oral health promotion campaigns and developing resources specific to Indigenous people, there seems to be a need for intensive person-centred behavioural interventions targeting both parents and children, as parents play a critical role in making food choices for children and in encouraging good oral hygiene [54].

In Australia, 80% of public water supplies are fluoridated. However, this does not include many regional and rural areas [55, 56]. The water was fluoridated in this community between ~ 2005 and ~ 2011, and the benefits of this are clear [56]. However, the fluoridation plant was damaged in 2011 and has not been repaired since: re-implementation is desirable.

Limitations: Conducting research in small and very remote settings comes with many challenges. Receiving consent is a complex process and communities may feel overwhelmed by the process. Even though we recruited a community member to assist with this process a significant number of parents whose children were invited to participate did not consent; and of those who consented a number could not provide a saliva specimen. A sample of almost 300 children, comprising half of all children, is a significant sample. However, because of the modest sample size, explanatory and confounding variables were dichotomised, and some had missing data, reducing the sample in the multivariate analysis. Oral health practices and soft drink consumption are self-reported, with possible over- or under-estimation a reality. The explanatory salivary characteristics variables were, however, measured objectively.

Cross-sectional design does not allow causation and temporality to be assessed. Our longitudinal data will, however, permit more confident evaluation of these parameters. A significant strength is that this, to our knowledge, is the first study to report these relationships from a remote Indigenous community in Australia, and perhaps even globally. Thus, although the sample size is modest, the findings might be applicable to remote Indigenous communities worldwide.

## Conclusions

Children with higher loads of salivary MS and LB experienced a greater burden of dental caries. In addition, less frequent tooth brushing and daily consumption of soft drinks was associated with greater caries experience. These observations will inform our approach to disease prevention in this and similar communities.

## Abbreviations

CFU/ml: Colony forming units per millilitre
CRT: Caries risk test
dmfs: Decayed, missing and filled deciduous tooth surface
DMFS: Decayed, missing and filled permanent tooth surface
dmft: Decayed, missing and filled deciduous teeth
DMFT: Decayed, missing and filled permanent teeth
ICDAS-II: International Caries Detection and Assessment system
IQR: Interquartile range
LB: Lactobacilli
MS: Mutans streptococci
